# The emerging role of histone acetylation in rheumatic diseases: unraveling mechanisms and therapeutic prospects

**DOI:** 10.3389/fimmu.2026.1741730

**Published:** 2026-02-25

**Authors:** Jianting Wen, Jian Liu, Lei Wan, Fanfan Wang, Yang Li

**Affiliations:** 1 Department of Rheumatology and Immunology, First Affiliated Hospital of Anhui University of Chinese Medicine, Hefei, Anhui, China; 2 Institute of Rheumatology, Anhui Academy of Chinese Medicine, Hefei, Anhui, China; 3 Anhui Province Key Laboratory of Modern Chinese Medicine Department of Internal Medicine Application Foundation Research and Development, Hefei, Anhui, China

**Keywords:** epigenetic modification, histone acetylation, mechanisms and therapeutic prospects, rheumatic diseases, traditional Chinese medicine

## Abstract

Histone acetylation, a fundamental and reversible epigenetic modification, critically regulates chromatin structure and plays a pivotal role in diverse cellular processes, including gene transcription, cell cycle progression, and DNA damage repair. Rheumatic diseases [such as rheumatoid arthritis (RA), osteoarthritis (OA), and systemic lupus erythematosus (SLE)] represent a group of disorders primarily affecting joints, bones, muscles, and connective tissues, posing a significant global burden. This review systematically elucidated the aberrant regulation of histone acetylation in these rheumatic conditions, with a focus on the disrupted balance between histone acetyltransferases (HATs) and histone deacetylases (HDACs). We further discussed the downstream mechanisms driven by these acetylation alterations, which contribute to various pathological processes (including synovial inflammation, cartilage degradation, and dysregulated cell death). Notably, special emphasis is placed on the potential of traditional Chinese medicine (TCM) to target this epigenetic axis. We summarized evidence that TCM formulations can exert their therapeutic effects by modulating HAT/HDAC activity and restoring acetylation homeostasis. By integrating current knowledge, this review aimed to provide mechanistic insights and highlight the promising potential of targeting histone acetylation, especially through TCM-based strategies, for developing novel therapeutic strategies against rheumatic diseases.

## 1 Introduction

Rheumatic diseases encompass a heterogeneous group of chronic inflammatory and degenerative conditions that primarily affect the joints, muscles, bones, and connective tissues, mostly including rheumatoid arthritis (RA), osteoarthritis (OA), and systemic lupus erythematosus (SLE) (1). These disorders are characterized by persistent pain, progressive functional impairment, and a substantially elevated risk of disability, all of which profoundly compromise patients’ quality of life (2, 3). Beyond the individual burden, disease management imposes a formidable economic burden on patients, healthcare systems, and society at large (4). Currently, the mainstream therapeutic strategies [including non-steroidal anti-inflammatory drugs (NSAIDs), conventional disease-modifying anti-rheumatic drugs (DMARDs), and biologic agents] aim to control symptoms and modify disease progression (5, 6). However, their efficacy is often incomplete, and their long-term use may be accompanied by significant adverse effects, especially hepatorenal toxicity and gastrointestinal complications (7). Hence, the existing therapeutic arsenal remains insufficient for a considerable number of patients, highlighting the urgent need to explore novel, safer, and more effective treatment modalities.

Despite extensive research, the precise etiologies of most rheumatic diseases remain incompletely understood, reflecting a multifaceted interplay of genetic predisposition, environmental triggers, and immune dysregulation (8). Although genome-wide association studies have identified numerous rheumatic disease-associated susceptibility loci, these genetic variants collectively explain only a limited proportion of disease heritability and fail to fully account for the variability in disease onset, clinical heterogeneity, and progression. This phenomenon of “missing heritability” highlights the essential role of non-heritable factors (such as environmental exposures and epigenetic regulation) in disease pathogenesis. Notably, epigenetic mechanisms, which mediate dynamic and reversible regulation of gene expression without altering the underlying DNA sequence, have emerged as a crucial link bridging genetic risk, environmental factors, and aberrant immune responses (9, 10). It has been shown that processes such as DNA methylation, histone modifications, and non-coding RNA-associated regulation are profoundly influenced by environmental factors and, in turn, orchestrate critical immune cell functions (including pro-inflammatory cytokine production) (11). Therefore, epigenetic dysregulation is increasingly recognized as a key contributor to the chronic inflammation that underlies rheumatic diseases, establishing epigenetics as a promising frontier for novel therapeutic exploration. Among the pivotal epigenetic mechanisms, histone modifications constitute a fundamental layer of gene regulation that dynamically controls chromatin architecture and DNA accessibility (12). These post-translational histone modifications play a central role in regulating chromatin states and gene transcription in a highly dynamic manner (13). Of these, histone acetylation is particularly notable as a critical regulator of inflammatory gene expression (14). This dynamic process is orchestrated by HATs and HDACs, which promote chromatin relaxation and condensation, respectively (15). Importantly, aberrant histone acetylation has been robustly documented in specific rheumatic diseases, particularly RA and SLE, where epigenetic alterations are repeatedly observed in patient-derived immune cells and affected tissues. This dysregulated acetylation landscape, typically driven by an imbalance in HAT/HDAC expression or activity, directly facilitates the pathogenic inflammatory cascade and loss of immune tolerance in rheumatic diseases. Therefore, restoring acetylation homeostasis presents a compelling therapeutic strategy for rheumatic diseases (16).

Certainly, the multi-component and multi-target nature of traditional Chinese medicine (TCM) provides a unique approach for managing complex diseases like rheumatism (17). Emerging evidence has suggested that the therapeutic benefits of TCM (including its modulation of inflammatory responses and immune homeostasis) may be partly attributed to its ability to regulate histone acetylation (18, 19). Active compounds derived from various herbal formulations and their metabolites have been identified as potential modulators of HAT or HDAC activity. By correcting dysregulated histone acetylation at specific gene promoters [such as those involved in the expression of pro-inflammatory cytokines like interleukin 6 (IL-6) and tumor necrosis factor (TNF)-α, as well as genes related to NF-κB signaling], these TCM-derived agents have been shown to suppress inflammatory gene transcription in fibroblast-like synoviocytes (FLSs) and immune cells, thereby promoting the resolution of chronic inflammation. This epigenetic mechanism provides a scientific basis for the empirically established efficacy of TCM and positions it as a valuable resource for developing novel epigenetics-based therapeutics against rheumatic diseases.

By integrating current evidence, this review aimed to (1) elucidate the role histone acetylation dysregulation in the pathogenesis of rheumatic diseases, (2) highlight the potential of TCM in modulating these epigenetic alterations; and (3) discuss promising translational prospects for novel therapeutic strategies targeting histone acetylation in rheumatic diseases.

## 2 The regulatory machinery: HATs and HDACs in immunity and homeostasis

### 2.1 Protein acetylation: an overview of molecular mechanisms

Precise regulation of proteins is crucial for the proper functioning of organisms. Among various regulatory processes, reversible post-translational modifications (PTMs) represent a highly effective mechanism for controlling protein activity. A key advantage of PTMs lies in their regulation speed and energy efficiency compared to *de novo* protein synthesis.

**Figure 1:**
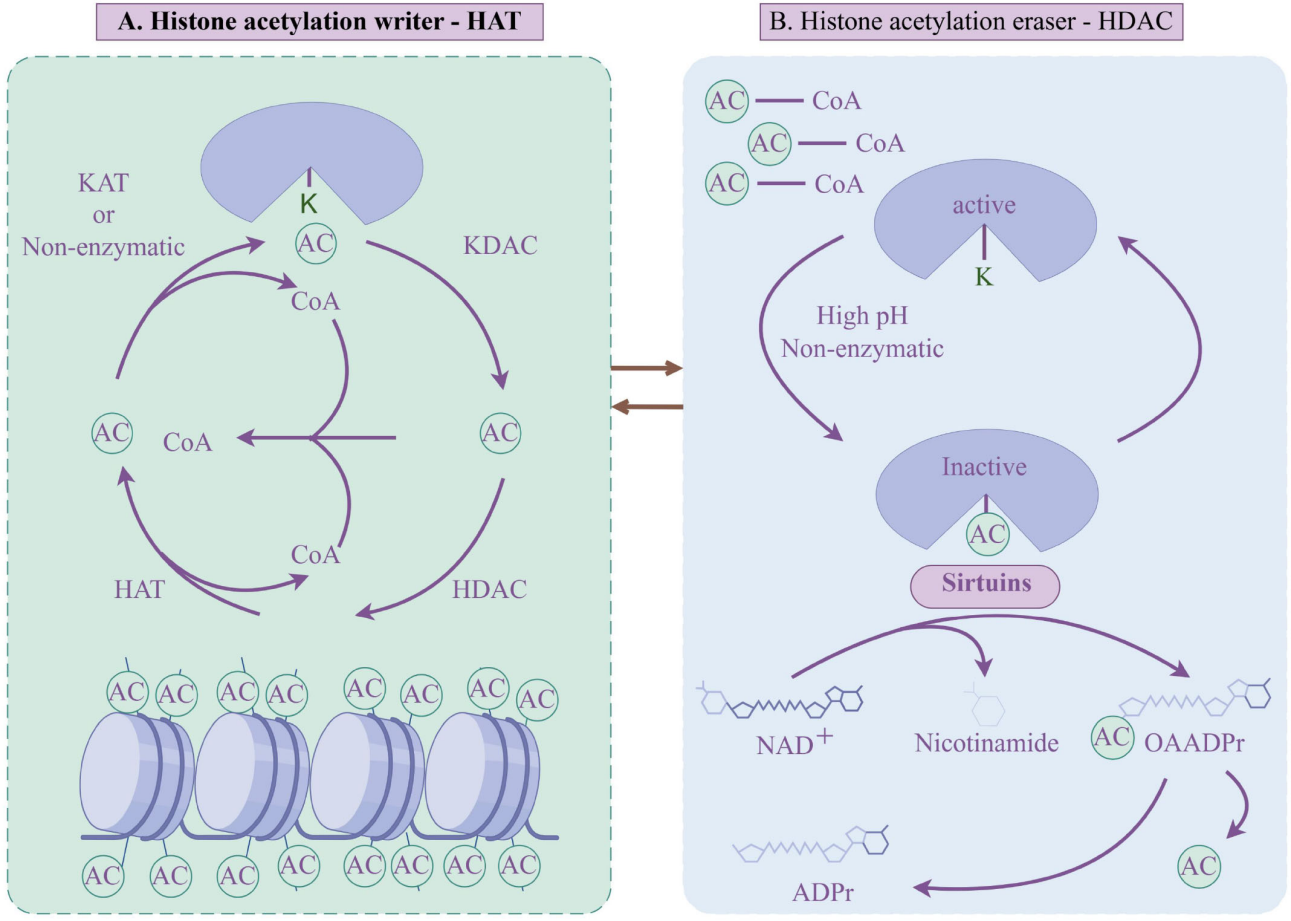
Mechanisms of histone acetylation regulation. **(A)** Histone acetylation “writers”: (HATs/KATs) catalyze the transfer of an AC from acetyl-CoA to lysine residues on histones. Some non-enzymatic mechanisms are also indicated. **(B)** Histone acetylation “erasers”: HDACs (including classical HDACs and NAD^+^-dependent sirtuins) remove ACs, regenerating the unmodified lysine. This reaction produces acetate (AC) or, in the case of sirtuins, nicotinamide and O-acetyl-ADP-ribose (OAADPr).

Lysine acetylation was first identified on histones by Vincent Allfrey and colleagues in 1964 (20). As an evolutionarily conserved PTM, acetylation occurs across both prokaryotes and eukaryotes (21). Subsequent discoveries (including the characterization of mammalian HATs and HDACs, the identification of acetyl-lysine reader domains, and the development of deacetylase inhibitors) collectively pave the way for studying non-histone protein acetylation (22). Over the past decade, mass spectrometry-based proteomics has significantly accelerated the identification and characterization of endogenous acetylated proteins, while also elucidating the regulatory mechanisms governing non-histone acetylation. At the molecular level, the dynamic balance of histone acetylation is directly regulated by the coordinated activities of HATs and HDACs. HATs catalyze the transfer of an acetyl group (AC) from acetyl-CoA to the ϵ-amino group of lysine residues on histone tails (23, 24). This acetylation neutralizes the positive charge of histones, thereby weakening the electrostatic interaction between the histones and the negatively charged phosphate groups of DNA. As a result, the chromatin structure transitions from a condensed to a more relaxed state, allowing enhanced accessibility for transcription factors and co-activators. Conversely, HDACs remove ACs from hyperacetylated histones, leading to a low acetylation state. This process promotes the transition of euchromatin to heterochromatin and is generally associated with gene silencing (**Figure 1**).

It is worth noting that HATs and HDACs regulate a broad range of substrates beyond histones, modifying numerous non-histone proteins as well. Therefore, some researchers have proposed renaming these enzymes as lysine acetyltransferases (KATs) and lysine deacetylases (KDACs) to better reflect their wider substrate specificity (25). However, given that this review focuses on epigenetic mechanisms, the conventional terms HAT and HDAC will be retained in subsequent sections to maintain clarity regarding their central role in regulating histone acetylation.

### 2.1 Classification of histone acetylation

Histone acetylation is dynamically regulated by the opposing actions of ‘writers’ (such as HATs) and ‘erasers’ (such as HDACs). A previous study has revealed that dysregulation or functional abnormalities of HATs and HDACs are implicated in the pathogenesis of various diseases, particularly cancer progression (26). Such disturbances often lead to widespread epigenetic alterations and aberrant transcriptional programs of critical genes. Given their central role in maintaining epigenetic homeostasis, these regulatory factors have emerged as promising targets in epigenetic-based therapeutics.

#### 2.1.1 Histone acetylation writer-HAT

Based on subcellular localization, HATs are categorized into two types: Type A and Type B (27). Specifically, Type A HATs are localized within the nucleus, whereas Type B HATs are primarily found in the cytoplasm. Type A HATs are responsible for acetylating histones within the context of chromatin. Similar to other histone-modifying enzymes, Type A HATs often function as part of multi-protein complexes, in which individual components play critical roles in regulating enzyme recruitment, catalytic activity, and substrate specificity (28). Type A HATs comprise several distinct families: (1) the GNAT family, including members such as PCAF (KAT2B), GCN5 (KAT2A), and ELP3; (2) the CBP/p300 family, comprising CBP (KAT3A) and p300 (KAT3B). Enzymes in this family exhibit broader substrate specificity and higher catalytic efficiency compared to other HATs, demonstrated by their ability to acetylate all core histone types as well as nucleosomes; (3) the MYST family, including Tip60 (KAT5), MOZ (KAT6A), MORF (KAT6B), HBO1 (KAT7), and MOF (KAT8); (4) the transcription factor-associated HAT family, encompassing TAFII250 (KAT4) and TIFIIIC90 (KAT12); and (5) several nuclear receptor coactivators, such as p600 (KAT13C), SRC1 (KAT13A), CLOCK (KAT13D), and NCOA3 (KAT13B).

**Figure 2:**
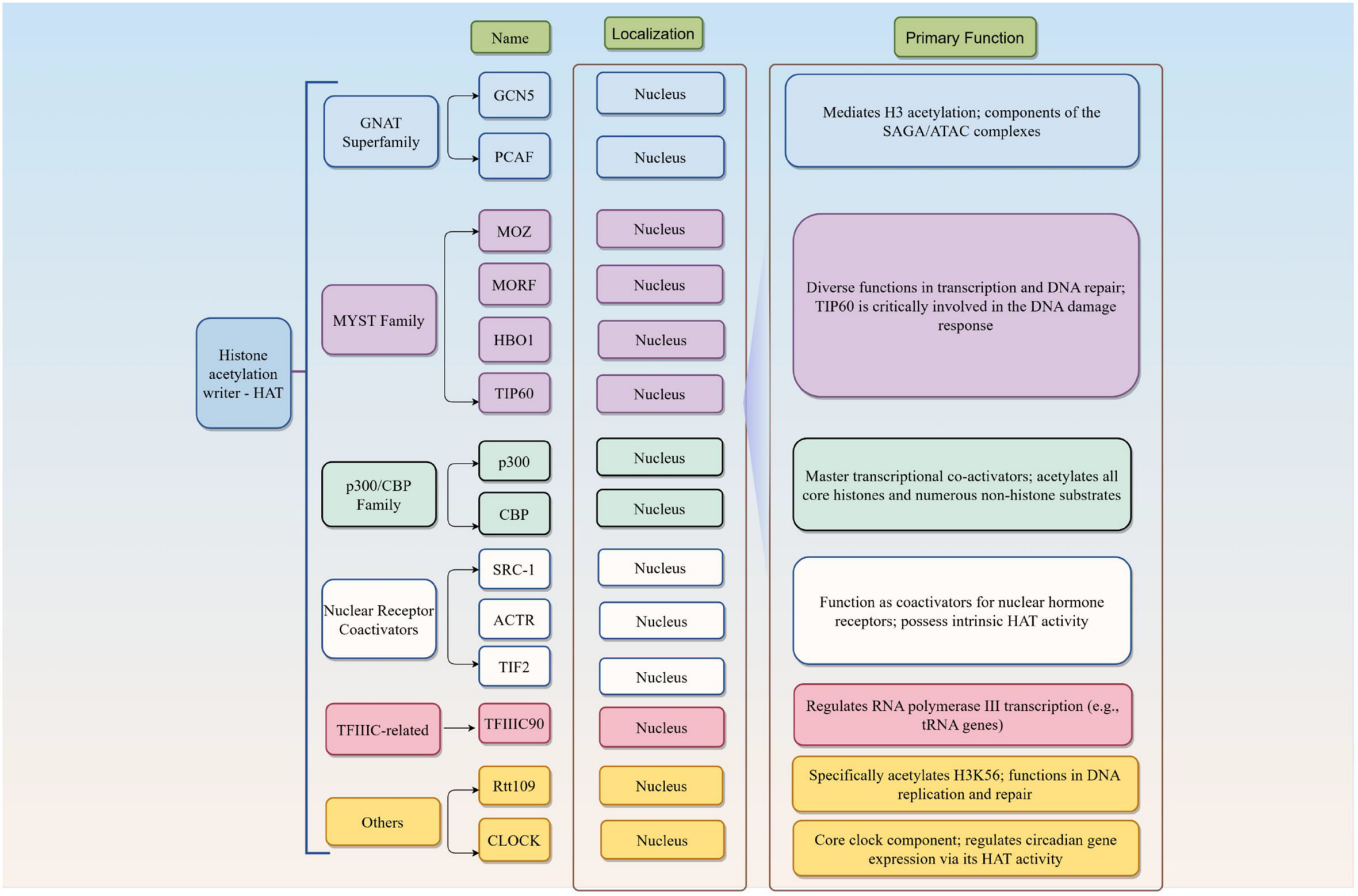
Subcellular localization and biological functions of HAT.

In contrast to their nuclear counterparts, Type B HATs specifically catalyze the acetylation of free histones within the cytoplasm. These enzymes are responsible for acetylating newly synthesized histone H4 at conserved lysine residues (primarily K5 and K12), as well as specific sites on histone H3. This distinctive acetylation pattern is essential for proper histone deposition and nucleosome assembly of histones into chromatin. Following incorporation, these specific acetyl marks are typically erased. Type B HATs are highly conserved, with all known members sharing sequence homology with the yeast Hat1 protein. Currently, the identified mammalian Type B HATs include HAT1 (KAT1) and HAT4 (NAA60) (**Figure 2**).

#### 2.1.2 Histone acetylation eraser-HDAC

The core function of HDACs is to catalyze the reverse reaction of acetylation, known as deacetylation (29). Based on their sequence homology to yeast deacetylases, HDACs are classified into two major families. The first family requires Zn²^+^ as an essential cofactor for catalytic activity and encompasses Class I (HDAC1, 2, 3, and 8), Class II (HDAC4, 5, 6, 7, 9, and 10), and Class IV (HDAC11). Class II enzymes are further subdivided into Class IIa (HDAC4, 5, 7, 9) and Class IIb (HDAC6, 10) based on their domain architecture. The second family, Class III HDACs, relies on NAD^+^ as an essential cofactor and is commonly referred to as sirtuins (30). Although individual HDACs often exhibit relatively low intrinsic substrate specificity and can catalyze deacetylation at multiple histone sites, they generally achieve functional specificity within cells by forming distinct multi-protein complexes. These complexes direct HDACs to specific genomic locations and regulate their activity toward particular substrates.

**Figure 3:**
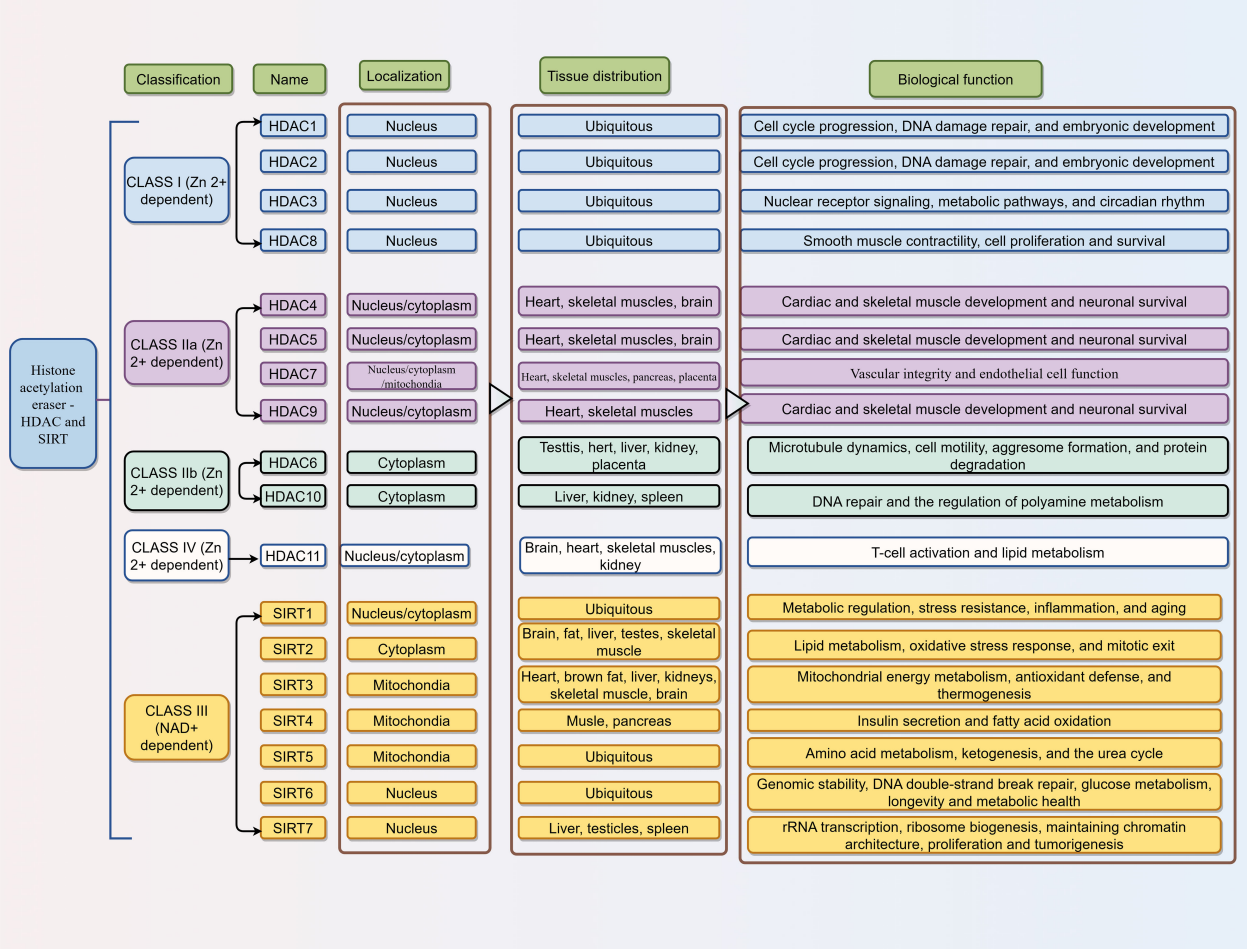
Subcellular localization, tissue distribution, and biological functions of HDAC and SIRT proteins across different classes. The figure summarizes mammalian HDACs, categorized into classes based on sequence homology and cofactor dependency. Classes I, II, IV, V, VI, and VII are Zn²^+^-dependent enzymes, whereas Class III comprises NAD^+^-dependent sirtuins (SIRT1-7). For each HDAC, the subcellular localization, tissue distribution, and key biological functions are listed, highlighting their diverse roles in cellular processes, development, and metabolism.

Class I HDACs, which share high sequence homology with yeast Rpd3, are characterized by a single, highly conserved deacetylase domain. Predominantly localized in the nucleus, they exhibit potent deacetylase activity toward histones. Class II HDACs are defined by their homology to yeast Hda1, which are characterized by a conserved C-terminal catalytic deacetylase domain. Unlike Class I enzymes, Class II HDACs can shuttle between the nucleus and cytoplasm, though they are often found in the cytoplasm, a pattern regulated by specific cellular signals. Class III HDACs, or sirtuins, are homologous to yeast Sir2 and represent an evolutionarily conserved protein family found from bacteria to humans. In mammals, 7 sirtuin isoforms (SIRT1-7) have been identified, each with distinct subcellular localizations: primarily in the nucleus (SIRT1, SIRT6, and SIRT7), cytoplasm (SIRT1 and SIRT2), or mitochondria (SIRT3, SIRT4, and SIRT5). Class IV HDAC, currently represented solely by HDAC11 (homologous to yeast Hos3), has been implicated in various cellular processes (**Figure 3**).

#### 2.1.3 Readers of protein acetylation

In addition to the “writers” and “erasers” of acetylation, the functional impact of protein acetylation also depends on specialized “readers,” which recognize acetylated lysine residues and translate these marks into downstream regulatory outcomes (31). Acetylation readers typically contain conserved structural domains that bind acetyl-lysine motifs, enabling the recruitment of transcriptional machinery or chromatin-remodeling complexes.

Among the well-characterized acetylation readers are proteins containing bromodomains, including members of the bromodomain and extraterminal domain (BET) family (32). By binding to acetylated histone tails, these readers serve as molecular scaffolds that link acetylation marks to transcriptional activation, enhancer assembly, and chromatin accessibility. In addition to histones, acetylation readers can recognize acetylated non-histone proteins, thereby modulating transcription factor activity, signal transduction, and inflammatory gene expression.

Importantly, growing evidence has implicated dysregulated acetylation readers in inflammatory and autoimmune diseases, highlighting their therapeutic potential. Pharmacological inhibition of bromodomain-containing readers can disrupt acetylation-dependent transcriptional programs without directly altering acetylation levels, offering a complementary strategy to targeting writers or erasers (33, 34). Taken together, writers, erasers, and readers constitute an integrated regulatory network that orchestrates the dynamic and context-dependent effects of protein acetylation.

## 3 Histone acetylation and rheumatic diseases

Histone acetylation critically regulates the pathogenesis of rheumatic diseases by modulating immune cell functions, inflammatory responses, and tissue homeostasis, as summarized in **Table 1**.

**Table 1:** Deregulated histone acetylation in rheumatic diseases.

Rheumatic diseases	Acetylated/deacetylated proteins	Targets	Mechanisms
RA	HDAC6	MMP-3, IL-6, CCL2, and CXCL8	Dampens the inflammatory and destructive activity of RA-FLS (47)
	SIRT1	NF-κB	Inhibites synovial hyperplasia and inflammation (48)
	HDAC5	IFNB, CXCL9, IRF1	Promotes nuclear localisation of IRF1 and transcription of a subset of type I IFN response genes (49)
	HDAC	IL-6	Suppresses inflammatory cytokine production in RA synovial macrophages and tissue (50)
	KAT2A	NLRP3	Inhibits inflammatory macrophage activation and RA through epigenetic and metabolic reprogramming (55)
	Sirt6	FOX1	Accelerates experimental RA by enhancing macrophage activation and infiltration into synovium (56)
	p300	lncRNA NEAT1/IL-18	LncRNA NEAT1 aggravated RA via p300/CBP/IL-18 axis (57)
	Tip60 (KAT5)	Foxp3	Impaired Tip60-mediated Foxp3 acetylation attenuates regulatory T cell development in RA (58)
	HDAC3	/	HDAC3 induces the increase in inflammation factors and participates in the pathogenesis of RA (59)
	SIRT1	AMPK	SIRT1/AMPKα signaling exerts anti-inflammatory activities by regulating M1/M2 polarization and reduces inflammatory responses in RA (61)
	HDAC1	miR-124/MARCKS-JAK/STAT	HDAC1 promotes synovial cell hyperplasia and synovial inflammation in mice with CIA by elevating miR-124 and MARCKS expression (62)
	HDAC4	miR-138/NF-κB/PGRN	MiR-138 regulates RA-related inflammatory cytokines in RA through HDAC4/PGRN or HDAC4/NF-κB (63)
	SIRT1	NF-κB	SIRT1 exacerbates inflammatory arthritis via the hyperactivation of NF-κB signaling (64)
	p300	cPLA2/MyD88/Akt/NF-κB	IL-1β-induced cPLA2 expression enhanced joint inflammatory responses in RA through activation of the MyD88/c-Src, MMP/HB-EGF, EGFR/PI 3-kinase/Akt, p300, and NF-κB pathways (65)
	SIRT1	p53/p65	SIRT1 participates in RA progression by promoting angiogenesis (68)
OA	SIRT1	p53	Relaxin-2 counteracts TNF-α-induced senescence in chondrocytes by enhancing telomerase activity and modulating SIRT1/p53 signaling (78)
	SIRT1	p21/IL-6R/JAK2	Targeting p21-positive senescent chondrocytes via IL-6R/JAK2 inhibition to alleviate OA (79)
	SIRT1	Ftl	Sirt1 overexpression inhibits chondrocyte ferroptosis via Ftl deacetylation to suppress the development of OA (81)
	SIRT1	miR-217-5p	MiR-217 regulates SIRT1 expression and promotes inflammatory and apoptotic responses in OA (83)
	SIRT1	PTEN/EGFR	SIRT1 represses the ubiquitination of EGFR by down-regulating PTEN, inhibits extracellular matrix degradation and activates chondrocyte autophagy, thereby performing an OA-alleviating role (85)
	SIRT3	MKK7	GADD45β-I attenuates oxidative stress and apoptosis via Sirt3-mediated inhibition of ER stress in OA chondrocytes (87)
	SIRT3	SOD2	Restoration of SIRT-3 in aging cartilage may improve cartilage resistance to oxidative stress by rescuing acetylation-dependent inhibition of SOD2 activity (88)
	HDAC3	miR-193b-3p	MiR-193b-3p directly targets HDAC3, promotes H3 acetylation, and regulates hMSC chondrogenesis and metabolism in PHCs (89)
	SIRT7	NDRG3/c-Raf/ERK	SIRT7 downregulates NDRG3 expression via directly reducing its acetylation and promoting its ubiquitination degradation, which consequently inhibits the c-Raf/ERK signaling pathway and suppresses osteoclast differentiation in OA (90)
	SIRT1	SOX9/P65	PGRN increased SIRT1 expression and activity, which reduced the acetylation levels of SOX9 and P65 and thereby promoted nuclear translocation of SOX9 and inhibited TNFα-induced P65 nuclear accumulation to maintain chondrocyte homeostasis (91)
	SIRT3	AMPK	AMPK activation, via SIRT3, limits oxidative stress and improves mtDNA integrity and function in OA chondrocytes (92)
	H3K27ac	LncRNA CRNDE/DACT1	LncRNA CRNDE promoted DACT1 expression through epigenetic modification and restrained the activation of Wnt/β-catenin signaling to impede the progression of OA (93)
SLE	H3K9	STAT3/RFX1	IL-6/STAT3 pathway induced deficiency of RFX1 contributes to Th17-dependent SLE via epigenetic regulation (106)
	H3 (Lys-18)	IL-17A, IL-17F	Extended involvement of CREMα in cytokine dysregulation in SLE by contributing to a disrupted balance between IL-17A and IL-17F (107)
	H3K4me3	TNFSF7	Aberrant histone modifications within the TNFSF7 promoter may contribute to the development of lupus by increasing CD70 expression in CD4(^+^) T cells (109)
	HDAC1	DNMT1/RFX1	Autoimmune responses in SLE are due in part to RFX1 downregulation in regulating the epigenetic status of T cells (110)
	H3K9	IL-17A, IL-17F	Increased expression of TLR2 contributes to immune reactivity and promotes IL-17A and IL-17F expression through histone modifications in SLE (108)
	p300	IRF1	Prolactin activates IRF1 and leads to altered balance of histone acetylation for SLE (111)
	H4	IRF1	IRF1 and histone H4 acetylation participate in SLE (112)
	H3	LncRNA IL21-AS1	LncRNA IL21-AS1 interacts with hnRNPU protein to promote IL21 overexpression and aberrant differentiation of Tfh cells in SLE (113)
	H3K27me3/H3K9/14ac	miR-1246/p53	The reduction of p53 decreases miR-1246 expression via upregulation of H3K27me3 and downregulation of H3K9/14ac, which in turn results in SLE B cell hyperactivity (114)
	H3K4me3	IL-2/IL-17A	Increased Set1 enrichment up-regulates H3K4me3 amount at the CREMα promoter, which antagonizes DNMT3a and suppresses DNA methylation within this region, consequently result in IL-2 under-expression and IL-17A overproduction, and contribute to SLE (115)

### 3.1 Deregulated histone acetylation in RA

RA is a systemic autoimmune disorder characterized by chronic synovitis, which often leads to joint deformities, with a global prevalence ranging from 0.2% to 1.0% and a predominance in females (35, 36). Clinically, RA typically presents with joint-related symptoms such as morning stiffness, swelling, and pain in the affected joints (37). As the disease progresses, these symptoms may result in joint deformity and impaired articular function. Additionally, some patients may also present with systemic symptoms such as fever and fatigue. Moreover, RA may also involve extra-articular organs, such as the lungs, heart, and hematopoietic system (38, 39). The pathogenesis of RA involves multiple interconnected mechanisms, including chronic inflammation, dysregulated apoptosis, oxidative stress, and pathological angiogenesis (40–42). FLSs and macrophages are key effector cells that drive synovial hyperplasia, inflammatory mediator production, and joint destruction. Notably, these pathogenic cellular and molecular processes are tightly regulated by epigenetic mechanisms, with histone acetylation playing a central role in controlling gene transcription and cellular activation. Proteomic analyses of protein acetylation in RA have revealed widespread alterations in protein acetylation. For instance, a study examining peripheral blood mononuclear cells (PBMCs) from RA patients has identified 29 lysine-acetylated protein spots (43). Among these, enolase 1 (ENO1) exhibits a markedly elevated acetylation level. This modification is regulated by HDAC1 and is hypothesized to contribute to RA pathogenesis by enhancing glycolysis-mediated energy supply, thereby sustaining lymphocyte activation and survival (43, 44).

#### 3.1.1 Synovial pathogenesis

FLSs are primary effectors in RA synovium, driving pathological synovial hyperplasia and inflammation (45). It has been shown that in RA, FLSs acquire an aggressively activated phenotype characterized by aberrant proliferation, migration, adhesion, invasion, and secretion of pro-inflammatory mediators (46). Thus, targeting these dysregulated functions represents a promising therapeutic strategy to suppress synovitis, prevent cartilage erosion, and ultimately mitigate disease progression.

Emerging evidence has revealed that the suppression of HDAC6 disrupts cytoskeletal dynamics, thereby attenuating FLS migration, invadopodia formation, and production of key cytokines, chemokines, and matrix-degrading enzymes (47). Mechanistically, inhibition of HDAC6 promotes α-tubulin acetylation, which effectively suppresses the pro-inflammatory and tissue-destructive functions of RA-FLSs. This highlights HDAC6 as a potential therapeutic target in RA.

Moreover, SIRT1 overexpression in RA-FLSs has been shown to inhibit cellular proliferation, migration, invasion, and pro-inflammatory cytokine secretion by inhibiting the NF-κB signaling through the deacetylation of the p65 subunit (48). These findings provide a clear mechanistic connection between non-histone protein acetylation and inflammatory regulation in RA synovial fibroblasts.

Furthermore, Angiolilli et al. have reported that pro-inflammatory cytokines (such as IL-1β and TNF) suppress HDAC5 expression in RA-FLSs (49). This epigenetic deregulation facilitates the nuclear translocation of IRF1 and subsequent transcription of interferon (IFN)-response genes, thereby amplifying local inflammatory response. Their work underscores that cytokine-mediated suppression of HDAC5 may be a key mechanism linking aberrant protein acetylation to synovitis, highlighting HDAC5 as a potential therapeutic target for RA. Additionally, Grabiec et al. have indicated that HDACi significantly reduce IL-6 production induced by pro-inflammatory cytokines in RA synovial fibroblasts and macrophages (50). Notably, this effect is not primarily mediated by alterations in the NF-κB or AP-1 signaling, but rather via a novel mechanism involving enhanced IL-6 mRNA decay. Taken together, these observations indicate that enhanced acetylation via HDAC inhibition can destabilize transcripts of key inflammatory cytokines, thereby attenuating synovial inflammation.

Collectively, current evidence supports the potential of targeting specific HDACs to modulate FLS activation and inflammatory signaling cascades in RA.

#### 3.1.2 Immune cell dysfunction

As a vital component of the immune system, T cells and macrophages play central roles in inflammatory response (51–53). Research has indicated that macrophages influence RA pathogenesis through multiple signaling pathways and metabolic mechanisms (54). Their polarization and function in RA are critically regulated by histone acetylation. KAT2A is a key acetyltransferase, which drives RA pathogenesis by orchestrating metabolic-epigenetic crosstalk in macrophages (55). It promotes H3K9 acetylation at pro-inflammatory gene promoters while suppressing NRF2-mediated antioxidant signaling, thereby facilitating glycolytic reprogramming and NLRP3 inflammasome activation. Additionally, Woo et al. have confirmed that myeloid SIRT6 deficiency exacerbates RA by promoting macrophage migration and synovial infiltration (56). Mechanistically, SIRT6 loss enhances the acetylation and stability of FoxO1, which drives pro-inflammatory responses. This underscores the critical role of SIRT6-mediated deacetylation in restraining macrophage-driven synovitis, positioning SIRT6 as a potential therapeutic target for RA. Additionally, Guo et al. have elucidated that long non-coding RNA (lncRNA) NEAT1, induced by p-p65, interacts with HAT p300 to epigenetically upregulate IL-18 expression via promoter histone acetylation; this NEAT1/p300 axis promotes synovial infiltration of macrophages and CD4^+^ T cells, thereby exacerbating RA pathogenesis (57). Dysregulated T cell function is also pivotal in RA, with lineage commitment governed by epigenetic mechanisms. Research has demonstrated that deficient expression of the HAT Tip60 in T cells from RA patients impairs acetylation of the transcription factor Foxp3, compromising its transcriptional activity and stability (58). This critical PTM defect disrupts the Treg/Th17 differentiation balance, promoting pro-inflammatory Th17 responses while impairing immunosuppressive Treg function, ultimately exacerbating RA synovial inflammation and autoimmune pathology.

PBMCs serve as crucial mediators and biomarkers in RA pathogenesis, reflecting systemic immune dysregulation and contributing to inflammatory cascades through cytokine production and T cell activation. Li et al. have revealed a significant imbalance in epigenetic regulation within RA PBMCs, characterized by reduced class I HDAC expression and activity (particularly HDAC3), along with enhanced HAT activity (59). This dysregulation leads to global histone H3 hyperacetylation, establishing a pro-inflammatory epigenetic landscape that may facilitate aberrant T cell activation and differentiation, thereby contributing to RA immunopathogenesis.

#### 3.1.3 Key signaling nexus

Histone acetylation serves as a crucial epigenetic modulator in RA by orchestrating the transcriptional activity of multiple pivotal signaling pathways (including NF-κB, AMPK, and JAK-STAT), thereby contributing significantly to disease pathogenesis and progression (60). At the chromatin level, histone acetylation primarily regulates the accessibility of promoters and enhancers of inflammation-related genes, thereby shaping the transcriptional landscape that supports sustained inflammatory signaling and pathogenic cell activation. For instance, Park et al. have found that SIRT1 exerts anti-arthritic effects by modulating macrophage M1/M2 polarization. Specifically, it promotes AMPKα phosphorylation and M2-associated gene expression, while concurrently suppressing NF-κB-mediated M1 polarization through deacetylation of histone substrates and inflammatory gene promoters (61). Another study has revealed that HDAC1 overexpression mediates epigenetic repression of miR-124 and MARCKS via promoter deacetylation (H3/H4), leading to JAK/STAT pathway activation that exacerbates synovial hyperplasia and inflammation in RA (62). Thus, HDAC1 inhibition can restore acetylation homeostasis and modulate this critical pathogenic axis, positioning acetylation modification as a central regulator of RA progression.

In parallel, non-histone acetylation represents a distinct but interconnected regulatory layer. Accumulating evidence has demonstrated that acetylation of key transcription factors-most notably the NF-κB p65 subunit-serves as a critical post-translational mechanism controlling inflammatory gene expression in RA. For example, a previous study has demonstrated that miR-138 promotes RA inflammation by targeting HDAC4, leading to histone H3 hyperacetylation and dysregulated NF-κB signaling, along with downregulated PGRN expression and enhanced the release of pro-inflammatory cytokines from RA-FLSs (63). Furthermore, Hah et al. have provided direct genetic evidence that myeloid-specific SIRT1 deficiency exacerbates RA by inducing hyperacetylation of the NF-κB p65 subunit (64). Unlike histone acetylation, p65 acetylation directly enhances NF-κB transcriptional activity, nuclear retention, and target-gene selectivity, thereby promoting M1 macrophage polarization, pro-inflammatory cytokine production, and osteoclastogenesis.

Notably, current evidence supports a coordinated relationship between histone acetylation and p65 acetylation in RA macrophages and synovial cells. Histone acetylation establishes a permissive chromatin environment for NF-κB-responsive genes, whereas p65 acetylation fine-tunes transcriptional amplitude and signal duration. Supporting this hierarchical interplay, Chi et al. have observed that IL-1β upregulates cPLA2 expression and PGE2 production via the PI3K/Akt pathway, which phosphorylates and recruits the HAT p300; this leads to targeted histone acetylation at the cPLA2 promoter and cooperative activation with NF-κB, thereby amplifying RA synovial inflammation (65). Together, these findings indicate that histone acetylation and non-histone acetylation function as complementary regulatory layers converging on NF-κB signaling to shape inflammatory gene programs in RA, though their precise synergistic or antagonistic relationships in specific pathological contexts require further elucidation.

All in all, histone acetylation emerges as a central epigenetic hub coordinating inflammatory signaling, cellular metabolism, and FLS activation in RA. Future studies focusing on disease- and cell-specific acetylation events at both chromatin and transcription-factor levels may facilitate the identification of more precise epigenetic targets for therapeutic intervention in RA.

#### 3.1.4 Angiogenesis

Angiogenesis has been extensively studied and confirmed as a critical component in the pathophysiological process of RA. Angiogenesis is closely associated with pathological changes such as inflammation, synovial hyperplasia, and bone destruction (66). As has been evidenced previously, angiogenesis contributes significantly to disease progression, and its inhibition can effectively slow RA progression (67). Leblond et al. have delineated a novel mechanism where decreased SIRT1 expression in RA endothelial cells (ECs) disrupts acetylome homeostasis, thereby exacerbating pathological angiogenesis (68). Endothelial SIRT1 deficiency promotes acetylation of key signaling molecules (including p53 and the NF-κB subunit p65), driving a pro-inflammatory, hyper-proliferative EC phenotype and upregulating the pro-angiogenic factor CYR61. This SIRT1-dependent hyperacetylation circuit critically underlies the enhanced neovascularization observed in RA. Consequently, pharmacological activation of SIRT1 not only normalizes dysregulated acetylation and reverses the pathogenic EC phenotype but also ameliorates experimental arthritis. These findings position the restoration of SIRT1 activity as a promising therapeutic strategy to counteract angiogenesis in RA.

### 3.2 Deregulated histone acetylation in OA

OA is a prevalent degenerative joint disease characterized by progressive breakdown of articular cartilage, subchondral bone sclerosis, synovitis, and osteophyte formation, ultimately leading to pain and functional impairment (69, 70). OA pathogenesis extends beyond mere mechanical wear, involving a complex interplay of genetic predisposition, aging, obesity, and low-grade inflammation; all these factors collectively disrupt the balance between cartilage synthesis and degradation, mediated largely by catabolic enzymes such as MMPs and ADAMTSs (71, 72). Epigenetic mechanisms play a critical role in linking environmental risk factors with dysregulated gene expression in OA pathophysiology (73). For example, aberrant histone modifications (such as altered acetylation and methylation patterns) can modulate chromatin accessibility, further enhancing the transcription of matrix-degrading enzymes (74). Additionally, dysregulated non-coding RNAs function as crucial post-transcriptional regulators, contributing to the sustained pathological state in OA; for instance, cartilage-protective miR-552-3p is downregulated, whereas pro-inflammatory lncRNAs (e.g., lncRNA PVT1) are upregulated (75). These reversible epigenetic alterations represent a central mechanism underlying the persistent, dysregulated phenotype of OA.

#### 3.2.1 Chondrocyte homeostasis

Recent research has highlighted the central role of cellular senescence in driving OA pathology (76). Senescent cells accumulate in aging and stressed joint tissues, where they disrupt tissue homeostasis through the persistent release of a robust senescence-associated secretory phenotype (SASP) (77). At the molecular level, the initiation and maintenance of the senescent phenotype are governed by intricate epigenetic controls, among which histone acetylation emerges as a key modulator. This PTM alters chromatin architecture to activate the transcription of senescence-driving and SASP-related genes. In a mechanistic study, relaxin-2 has been shown to counteract TNF-α-induced chondrocyte senescence, a key process in OA pathogenesis (78). This protective effect was linked to the modulation of protein acetylation, a critical PTM in cellular aging. Specifically, relaxin-2 upregulates SIRT1, which reduces the acetylation of p53 at K382, thereby attenuating the senescent phenotype. This finding highlights the acetylation status of proteins such as p53 as a pivotal regulatory hub in senescence. Moreover, another study has elucidated a novel mechanism whereby JAK2 inhibition alleviates OA by stabilizing SIRT1, leading to reduced acetylation of the essential chondrogenic transcription factor SOX9 (79). This decrease in SOX9 acetylation facilitates its nuclear localization, restoring cartilage homeostasis. Furthermore, the intervention promotes clearance of p21-positive senescent cells, directly linking the regulation of protein acetylation to the amelioration of cellular senescence in OA.

Additionally, ferroptosis also contributes to OA pathogenesis (80). Histone acetylation acts as a key epigenetic regulator of this process by modulating the expression of core ferroptosis-related genes in chondrocytes. As indicated by a seminal study by Xiong et al., the deacetylase SIRT1 plays a protective role by directly deacetylating the ferritin light chain (Ftl) at its K181 residue (81). This PTM stabilizes Ftl, enhancing cellular iron storage capacity and thereby suppressing ferroptosis in chondrocytes. These findings, validated *in vivo*, establish protein deacetylation as a crucial mechanism that negatively regulates ferroptosis, highlighting the SIRT1-Ftl axis as a promising therapeutic target for mitigating OA progression.

Histone acetylation further orchestrates both inflammatory and apoptotic responses, synergistically driving OA progression (82). In support of this, Papageorgiou et al. have observed a critical microRNA-epigenetic axis in OA pathogenesis, demonstrating that miR-217-5p-induced downregulation of SIRT1 leads to hyper-acetylation of the NF-κB p65 and p53 transcription factors (83). This PTM potentiates their transcriptional activity, increasing expression of pro-inflammatory cytokines (e.g., IL-1β, IL-6, and TNF-α) and pro-apoptotic factors (e.g., Bax), which collectively promote cartilage destruction.

As an epigenetic driver, histone hyperacetylation also dysregulates chondrocyte autophagy, thereby accelerating OA progression (84). According to a study by Lu et al., the regulatory role of acetylation in chondrocyte autophagy is evidenced by the SIRT1-PTEN-EGFR axis (85). Specifically, SIRT1-mediated deacetylation of PTEN reduces its stability, subsequently diminishing EGFR ubiquitination and enhancing its expression, ultimately activating protective autophagy and alleviating OA progression.

Moreover, histone hyperacetylation epigenetically dysregulates the antioxidant response, exacerbating oxidative stress and promoting OA (86). Zhang et al. have identified a novel cytoprotective axis in OA involving the MKK7 inhibitor GADD45β-I, which upregulates the mitochondrial deacetylase SIRT3; SIRT3 then deacetylates and activates the key antioxidant enzyme SOD2 (87). This PTM enhances mitochondrial antioxidant capacity, effectively alleviates IL-1β-induced oxidative stress and the associated endoplasmic reticulum stress in chondrocytes, thereby mitigating apoptosis. Alternatively, a recent study by Fu et al. has revealed that age-related loss of the deacetylase SIRT-3 leads to hyperacetylation of SOD2, which inhibits its antioxidant activity, thereby exacerbating oxidative damage and ultimately promoting OA pathogenesis (88).

#### 3.2.2 Cartilage metabolism and transcription

Osteoarthritic cartilage degeneration involves dysregulated metabolism and transcription, governed by complex signaling networks and epigenetically modulated through histone acetylation. Meng et al. have demonstrated that miR-193b-3p promotes chondrogenesis and cartilage matrix homeostasis by directly targeting HDAC3, thereby enhancing histone H3 acetylation at promoters of key anabolic genes (e.g., COL2A1 and ACAN) and suppressing catabolic pathways. This epigenetic mechanism underscores the critical role of acetylation in regulating cartilage metabolism (89). Another study has revealed that SIRT7, which may be downregulated in OA, inhibits OA osteoclast differentiation by directly binding to and deacetylating NDRG3, thereby promoting its ubiquitin-mediated degradation. This process attenuates the downstream c-Raf/ERK signaling cascade, highlighting a novel epigenetic-metabolic axis in OA pathogenesis (90). Furthermore, progranulin (PGRN) has been shown to maintain chondrocyte homeostasis in OA by upregulating SIRT1; SIRT1 subsequently deacetylates SOX9 to enhance its anabolic function and deacetylates P65 to suppress NF-κB-mediated catabolism. These findings underscore the pivotal role of SIRT1-dependent deacetylation in mediating the chondroprotective effects of PGRN (91).

Chen et al. have delineated an AMPK/SIRT3 axis crucial for mitochondrial integrity in OA chondrocytes (92). Specifically, AMPK activation enhances SIRT3 activity, which deacetylates and activates the mitochondrial antioxidant SOD2 and DNA repair enzyme OGG1; this coordinated action mitigates oxidative stress, restores mtDNA integrity, and promotes chondroprotection. Similarly, Zhang et al. have confirmed that lncRNA CRNDE exerts chondroprotective effects by epigenetically upregulating DACT1 (93). CRNDE recruits the HAT p300 to catalyze H3K27ac enrichment at the DACT1 promoter, thereby suppressing the Wnt/β-catenin signaling. This further highlight the crucial role of histone acetylation in mitigating OA progression.

### 3.3 Deregulated histone acetylation in SLE

SLE is a chronic and multifaceted autoimmune disease characterized by a breakdown of immune tolerance, leading to the production of diverse autoantibodies and subsequent multi-organ damage (94). Clinically, SLE manifests with significant heterogeneity, commonly involving the skin, joints, kidneys, and hematological system (95). SLE pathogenesis arises from a complex interplay among genetic susceptibility, hormonal influences, and environmental triggers, which collectively drive aberrant immune activation (96).

Emerging evidence indicates that epigenetic dysregulation, especially alterations in histone acetylation, plays an important role in immune imbalance in SLE (97, 98). Increased HAT activity coupled with reduced HDAC function has been associated with histone hyperacetylation at promoters of immune-related genes, thereby facilitating sustained transcription of inflammatory and autoimmune pathways. This link between environmental signals and gene expression regulation highlights histone acetylation as a context-dependent epigenetic regulator and a potential therapeutic target in SLE.

#### 3.3.1 Global hypoacetylation

B lymphocytes are critically implicated in the pathogenesis of SLE (99). Global alterations in histone acetylation observed in these cells highlight the significance of histone modifications in SLE B cells (100). In SLE patients, B lymphocytes exhibit marked global hypoacetylation of histones H3 and H4, underscoring impaired histone acetylation as a key epigenetic lesion in these cells. Monocytes are also pivotal in SLE pathogenesis, contributing to disease progression through aberrant cytokine production and self-antigen presentation. In support of this, a previous study has revealed significant alterations in H4ac in SLE monocytes (101). This hyperacetylation is predominantly linked to an IFN signature, with IRF1 binding sites enriched upstream of genes exhibiting concurrent increases in both H4ac and expression. These findings underscore the critical role of IFN-driven epigenetic remodeling via histone acetylation in modulating monocyte function in SLE.

Advances in proteomics have revealed a growing number of acetylated proteins. A recent study has further elucidated the role of epigenetic modifications in SLE, identifying CDCA5 and MCTS1 as potential biomarkers whose pathogenesis may be co-regulated by lactylation and acetylation, highlighting the interplay between these modifications in SLE immune dysregulation (102). Additionally, serum glycoprotein acetylation (GlycA) is significantly elevated in active SLE (103). Notably, GlycA independently predicts the severity of proliferative lupus nephritis, highlighting the potential role of protein acetylation in SLE pathogenesis and renal involvement. Similarly, another study has identified significant dysregulation of HDACs in SLE, with HDAC1 upregulated and HDAC2 downregulated in PBMCs (104). These alterations in acetylation-related enzyme expressions correlate with disease activity and duration, further linking epigenetic dysregulation to SLE progression.

#### 3.3.2 T cell hyperactivity

T cells contribute to SLE pathogenesis through aberrant activation and inflammatory cytokine production, processes that are critically regulated by histone acetylation (105). For instance, Zhao et al. have revealed that the transcription factor RFX1, downregulated in SLE CD4^+^ T cells, functions as a critical epigenetic brake on Th17 differentiation by modulating histone H3 acetylation at the IL-17A locus, thereby linking STAT3 signaling to dysregulated acetylation in autoimmunity (106). In contrast to IL-17A, IL-17F expression is suppressed in SLE T cells (107). This suppression is mediated by CREMα binding to its promoter, a repressive mechanism that operates independently of active histone acetylation at the locus. Additionally, another study has demonstrated that TLR2 overexpression in SLE T cells promotes pro-inflammatory cytokine production and Th17 responses by directly remodeling the epigenetic landscape of cytokine genes, including enhanced histone H4 acetylation at the IL-17A/F promoters (108).

Zhou et al. have elucidated that CD70 overexpression in SLE CD4^+^ T cells is driven by coordinated epigenetic alterations at the TNFSF7 promoter, including DNA hypomethylation and active histone modifications such as enhanced H3/H4 acetylation and H3K4 dimethylation. This reveals how histone acetylation collaborates with other epigenetic marks to dysregulate T cell function (109). Furthermore, Zhao et al. have identified RFX1 as a key epigenetic regulator in SLE; its downregulation disrupts the recruitment of HDAC1 and DNMT1 to target gene promoters, leading to histone hyperacetylation and DNA demethylation that drive aberrant expression of CD11a and CD70, thereby promoting autoimmunity (110).

#### 3.3.3 Innate immunity involvement

The innate immune system drives SLE progression through aberrant activation of pattern recognition receptors and immune cells, which instigate pathogenic inflammation and autoantibody production via epigenetic reprogramming and cytokine dysregulation. Leung et al. have proved that prolactin activates IRF1 and promotes its interaction with HATs CBP/p300 in monocytes, thereby linking hyperprolactinemia to the IFN signature in SLE (111). Further research has demonstrated that the transcription factor IRF1 directly interacts with HATs (including p300 and PCAF), driving pathologic H4 hyperacetylation in SLE monocytes. This establishes a mechanistic link between immune activation and chromatin modification in lupus pathogenesis (112).

As has been evidenced previously, lncRNA IL21-AS1 is upregulated in SLE CD4^+^ T cells and promotes T follicular helper (Tfh) cell differentiation (113). Mechanistically, IL21-AS1 forms a complex with hnRNPU to recruit the acetyltransferase CBP to the IL21 promoter, thereby epigenetically enhancing IL21 transcription via histone H3 acetylation. This pathway amplifies the germinal center response, identifying IL21-AS1 as a key regulator and a potential therapeutic target for SLE. Furthermore, in SLE B cells, underexpressed p53 recruits JMJD3 and EP300/CBP to the miR-1246 promoter, resulting in increased repressive H3K27me3 and decreased active H3K9/K14ac (114). This epigenetic silencing of miR-1246 promotes B cell hyperactivity and autoantibody production, revealing a novel p53-miR-1246 axis in SLE pathogenesis. Additionally, Zhang et al. have highlighted that epigenetic dysregulation of CREMα in SLE CD4^+^ T cells is primarily driven by Set1-mediated H3K4me3, while histone acetylation (H3ac/H4ac) levels remain unaltered (115). This underscores a specific mechanism in SLE where H3K4 hypermethylation, rather than acetylation, antagonizes DNMT3a to reduce DNA methylation, thereby enabling CREMα overexpression and the subsequent IL-2/IL-17A imbalance that drives pathogenesis.

## 4 TCM in treating rheumatic disease by targeting histone acetylation

In TCM, rheumatic diseases are classified as *“Bi Zheng”*, which describes conditions characterized by blockage of the meridians and collaterals. According to TCM theory, their pathogenesis involves complex and interconnected mechanisms such as deficiency of qi and blood, dysregulation of the nutritive and defensive systems, spleen–stomach weakness leading to endogenous dampness turbidity, and the subsequent interplay of phlegm retention and blood stasis. Rather than representing a single pathological entity, these processes collectively lead to chronic inflammation, tissue damage, and functional impairment, which conceptually parallels modern perspectives on immune dysregulation and persistent synovitis.

TCM demonstrates unique therapeutic potential in rheumatic diseases through the synergistic actions of multiple components, targets, and signaling pathways. Recent studies have increasingly suggested that the anti-rheumatic effects of TCM-derived compounds extend beyond symptom management and may involve epigenetic regulation, particularly the modulation of histone acetylation. By regulating HATs and HDACs, these compounds influence the transcription of genes associated with inflammatory responses, immune cell differentiation, and synovial proliferation, thereby providing a molecular basis for the holistic effects of TCM (summarized in **Table 2**). However, the mechanistic depth and translational relevance of these findings vary considerably, underscoring the need for more systematic and critical evaluation.

**Table 2:** TCM in treating rheumatic disease by targeting histone acetylation.

Rheumatic diseases	TCM	Acetylated/deacetylated proteins	Targets	Mechanisms
RA	Resveratrol	SIRT1	NLRP3	Resveratrol inhibits NLRP3 inflammasome activation by activating SIRT1, and significantly ameliorates arthritis in CIA models (118).
	Resveratrol	SIRT1	MAPK/NF-κB	Resveratrol up-regulates Sirt1 by inhibiting the interaction of AP-1 and NF-κB with COX-2 promoter in RA-FLS (119)
	Shikonin	H3	lncRNA-NR024118	Shikonin dose-dependently increases acetylation of histone H3 at the promoter of NR024118, inhibites inflammatory response and alleviate RA (120)
	Baicalin	SIRT1	NF-κB p65	Baicalin treatment can alleviate CIA in rats, by suppressing synovial NF-κB p65 protein expression and the elevation of its deacetylation by sirt1 (121)
	Cryptotanshinone	P300	STAT3	The anti-arthritis effects of Cryptotanshinone were attained through suppression of p300-mediated STAT3 acetylation (122)
	Wutou decoction	SIRT1	HMGB1/NF-κB	Wutou decoction attenuates RA in rats through SIRT1-mediated deacetylation of the HMGB1/NF-κB pathway (124)
	Wutou decoction	H3/H4	/	Wutou decoction may modulate H3 acetylation, functioning as anti-inflammatory potential in CIA (125)
	Qing Luo Yin	SIRT1	NF-κB	Qing Luo Yin eased AIA rats by inhibiting SIRT1-controlled visfatin production in white adipose tissues (126)
OA	Celastrol	SIRT2	NLRP3	Celastrol exerts its anti-inflammatory effects in OA by targeting the SIRT2-NLRP3 axis to inhibit chondrocyte pyroptosis (128)
	Resveratrol	SIRT1	NF-κB	Resveratrol inhibits IL-11β-mediated OA articular chondrocytes by activating SIRT1 and thereby suppressing nuclear factor-κB activity (129)
	Curcumin	SIRT3	SOD2-ROS	Curcumin regulates autophagy through SIRT3-SOD2-ROS signaling pathway to improve quadriceps femoris muscle atrophy in KOA rat model (130)

### 4.1 TCM monomers

Resveratrol, a natural polyphenolic compound, has been extensively studied as a potent activator of SIRT1, an NAD^+^-dependent class III HDAC (116). Its relationship with acetylation centers on its ability to modulate the deacetylation process (117). Cai et al. have reported that resveratrol ameliorates arthritis in collagen-induced arthritis (CIA) and citrullinated collagen–induced arthritis (Cit-CIA) models via dual mechanisms: (i) suppressing NLRP3 inflammasome activation via SIRT1-mediated deacetylation, and (ii) competitively binding to integrin α5β1 to inhibit ACPA-induced Pannexin/ATP signaling (118). Another study by Yang et al. has demonstrated that resveratrol, via SIRT1 activation, suppresses bradykinin-induced COX-2/PGE_2_ signaling in RA synovial fibroblasts (119). Mechanistically, this occurs via inhibition of the B2R-PKCμ-MAPK cascade and reduction in acetylation and promoter occupancy of NF-κB p65 and AP-1 (c-Jun/c-Fos), highlighting a non-histone acetylation–dependent epigenetic mechanism underlying its anti-arthritic effect.

Shikonin, the primary bioactive constituent of Lithospermum erythrorhizon roots, exhibits significant anti-inflammatory properties and modulates both cellular and humoral immune responses. These immunoregulatory activities are particularly implicated in its therapeutic potential against RA. A previous study has highlighted that shikonin promotes histone H3 acetylation at the promoter of lncRNA-NR024118, demonstrating anti-inflammatory effects (120). This epigenetic reprogramming suppresses the production of pro-inflammatory cytokines and matrix metalloproteinases in RA synovial fibroblasts, illustrating a promoter-specific histone acetylation mechanism rather than a global epigenetic mechanism of action.

Baicalin, a major flavonoid glycoside isolated from the roots of Scutellaria baicalensis Georgi (Huang Qin), exerts multifaceted therapeutic effects against RA through modulating pivotal inflammatory signaling pathways, attenuating immune dysregulation, and inhibiting synovial hyperplasia. Wang et al. have revealed that baicalin ameliorates CIA rats by upregulating SIRT1, which promotes deacetylation of NF-κB p65 (121). This PTM, along with reduced p65 phosphorylation, attenuates NF-κB transcriptional activity and downstream cytokine production, illustrating the functional interplay between acetylation and phosphorylation in RA inflammation.

Cryptotanshinone, a predominant quinoid diterpene isolated from the rhizomes of Salvia miltiorrhiza Bunge (Danshen), exhibits pleiotropic pharmacological activities. As a STAT3 inhibitor, cryptotanshinone responds to STAT3-induced cytokines and growth factors. Wang et al. have indicated that cryptotanshinone ameliorates arthritis in a CIA mouse model by correcting the Th17/Treg imbalance (122). This immunomodulatory effect is mediated through inhibition of p300-dependent STAT3 acetylation, a critical modification that cooperates with phosphorylation to regulate STAT3 transcriptional activity and Th17 differentiation, highlighting HAT-dependent control of immune cell fate.

### 4.2 TCM compounds

Wutou decoction, a classical TCM formula consisting of *Aconiti Radix*, *Ephedrae Herba*, *Paeoniae Radix Alba*, *Astragali Radix*, and *Glycyrrhizae Radix*, is traditionally used to dispel cold and dampness and relieve pain (123). In RA management, it exhibits multimodal actions, including alleviating joint inflammation, modulating immune dysregulation via suppressing pro-inflammatory cytokines, and providing analgesic effects, likely mediated by synergistic interactions among its bioactive constituents. Emerging evidence has implicated that the therapeutic effects of Wutou decoction in RA may involve the epigenetic regulation of histone acetylation. For example, Shen et al. have demonstrated that Wutou decoction ameliorates RA by upregulating SIRT1, which promotes deacetylation of HMGB1 and the NF-κB p65 subunit (124). This reduction in acetylation inhibits nuclear translocation and transcriptional activity of these proteins, leading to decreased cytokine production and M1 macrophage infiltration. Additionally, another study has revealed that Wutou decoction exerts anti-arthritic effects through dual epigenetic regulation: downregulation of DNMT1 leading to global DNA hypomethylation, alongside enhanced histone H3 acetylation in PBMCs (125). These findings indicate coordinated regulation across multiple epigenetic layers rather than isolated histone acetylation events.

Qingluo Yin, a multi-herbal formula containing *Cynanchum atratum*, *Lonicera japonica*, and *Rehmannia glutinosa*, is traditionally used to clear damp-heat and unblock meridians. According to pharmacological studies, Qingluo Yin suppresses pro-inflammatory cytokines (such as TNF-α and IL-6), inhibits the NF-κB signaling, and reduces synovial hyperplasia and angiogenesis. Wang et al. have reported that Qingluo Yin partially exerts its anti-rheumatic effects by inhibiting SIRT1 in white adipose tissue, resulting in a hyperacetylated state (126). This acetylation-dependent mechanism downregulates the expression of the pro-inflammatory adipokine visfatin, thereby linking systemic metabolic inflammation to joint pathology.

### 4.3 TCM in treating OA by targeting histone acetylation

Celastrol (CSL), a quinone methide triterpenoid isolated from *Tripterygium wilfordii* (Thunder God Vine), has long been used in TCM for treating inflammatory and autoimmune disorders (127). It exhibits broad pharmacological activities, including potent anti-inflammatory, immunosuppressive, antioxidant, and anti-tumor effects. Given its anti-inflammatory properties, CSL has emerged as a promising agent for OA treatment. A recent study has indicated that CSL mitigates OA progression by upregulating SIRT2, which directly promotes deacetylation of the NLRP3 inflammasome (128). This acetylation-dependent suppression inhibits NLRP3 activation and Gasdermin D-mediated pyroptosis in chondrocytes, thereby attenuating cartilage degeneration and joint inflammation.

Resveratrol has also been demonstrated to have chondroprotective effects in OA. As a SIRT1 activator, resveratrol enhances deacetylation of the NF-κB p65 subunit in chondrocytes (129). Specifically, deacetylation of Lys310 on p65 suppresses its nuclear translocation and transcriptional activity, leading to inhibition of IL-1β-induced iNOS expression and nitric oxide production, which collectively reduces inflammatory damage in OA.

Curcumin, a polyphenolic compound derived from *Curcuma longa* L., exhibits potent anti-inflammatory and antioxidant properties. As indicated by a recent study, curcumin ameliorates knee OA-associated quadriceps femoris atrophy by activating the SIRT3-SOD2 axis (130). SIRT3-mediated deacetylation of SOD2 enhances its antioxidant capacity, reduces reactive oxygen species accumulation, and suppresses excessive autophagy, thereby preserving muscle function and joint integrity.

Taken together, these findings indicate that distinct TCM-derived compounds modulate OA pathogenesis through activation of specific sirtuin-dependent deacetylation pathways. By targeting substrates such as NLRP3, NF-κB p65, and SOD2, these compounds converge on inflammasome regulation, inflammatory signaling, and oxidative stress control. However, most evidence remains at the preclinical stage. Challenges related to target specificity, dosage standardization, and long-term safety must be addressed prior to successful clinical translation.

## 5 Conclusions and perspectives

This review has systematically delineated the pivotal role of histone acetylation, a dynamic and reversible epigenetic modification, in the pathogenesis of rheumatic diseases (including RA, OA, and SLE). A key finding is the frequent imbalance between HATs and HDACs across these disorders. This imbalance drives a pathogenic cascade characterized by aberrant gene expression programs that sustain chronic inflammation, synovial hyperplasia, immune dysregulation, cartilage degradation, and aberrant cell death modalities (such as apoptosis, pyroptosis, and ferroptosis).

In RA, dysregulated acetylation orchestrates the activation of key effector cells (such as FLSs and macrophages), amplifies pro-inflammatory signaling through hubs like NF-κB and JAK-STAT, and promotes pathological angiogenesis. In OA, altered histone acetylation disrupts chondrocyte homeostasis, accelerating senescence, impairing autophagy, exacerbating oxidative stress and ferroptosis, and shifting cartilage metabolism toward catabolism. SLE is characterized by complex, cell-type-specific acetylation aberrations, ranging from global hypoacetylation in B cells to promoter-specific hyperacetylation in T cells and monocytes, which drive the breakdown of immune tolerance, autoantibody production, and systemic inflammation. These findings collectively underscore histone acetylation not merely as an associated phenomenon, but as a fundamental mechanistic driver and a convergent node that integrates genetic susceptibility and environmental triggers in rheumatic pathogenesis.

A particularly promising and innovative perspective highlighted in this review is the therapeutic potential of TCM in targeting this epigenetic machinery. Specifically, the multi-component, multi-target nature of TCM aligns well with the complex pathogenesis of rheumatic diseases. As summarized, numerous TCM-derived active compounds (e.g., resveratrol, baicalin, shikonin, cryptotanshinone, CSL, and curcumin) and formulated decoctions (e.g., Wutou decoction and Qingluo Yin) have demonstrated potent ability to ameliorate disease pathology by selectively modulating HAT/HDAC activity. Their mechanisms involve restoring acetylation homeostasis on critical substrates (such as NF-κB p65, STAT3, NLRP3, SOD2, and SOX9), thereby suppressing inflammatory cascades, rectifying immune cell imbalances, inhibiting synovial invasion, and preserving cartilage integrity. This provides a novel, epigenetically grounded scientific rationale for the empirically documented efficacy of TCM, positioning it as a rich resource for developing novel epigenetic-based therapeutics.

Looking forward, several key directions and challenges define the future perspective of this field. First, there is a pressing need to move beyond correlative studies and establish direct causal links between specific acetylation marks at precise genomic loci and functional outcomes in human rheumatic tissues. Advanced techniques such as CUT&Tag and base-editor screens will be instrumental in achieving this goal. Second, the cell-type and context-specific nature of acetylation effects demands greater emphasis. For example, the same HDAC or HAT can exert opposing functions in different cell types (e.g., SIRT1 in macrophages versus chondrocytes) or across disease stages. Future research should therefore employ cell-specific knockout models and single-cell multi-omics approaches to unravel this complexity, which is essential for developing targeted therapies with minimized off-target effects.

Third, the clinical translation of histone deacetylase inhibitors (HDACis) faces significant challenges related to limited target specificity and dose-limiting toxicities. Although pan- HDACis have shown anti-inflammatory efficacy in preclinical models, their broad activity across multiple HDAC isoforms and non-histone substrates often leads to off-target effects and adverse events, including hematological, gastrointestinal, and hepatic toxicities reported in clinical trials. These limitations underscore the need for next-generation epigenetic agents with improved isoform selectivity, optimized dosing strategies, and tissue- or cell-targeted delivery systems.

Fourth, despite their therapeutic promise, the clinical application of TCM-based epigenetic interventions is constrained by challenges in standardization, quality control, and batch-to-batch consistency. The complex and variable composition of herbal formulas complicates precise dosing, reproducibility, and pharmacokinetic evaluation. Addressing these issues through standardized manufacturing protocols, rigorous chemical profiling, and systematic pharmacological and toxicological assessment will be critical for advancing TCM-derived epigenetic modulators toward clinical use. In this context, TCM compounds, which often exhibit natural polypharmacology, may serve as valuable sources of inspiration and potential lead compounds for developing safer and more selective epigenetic therapeutics.

Finally, the acetylation of non-histone proteins, particularly metabolic enzymes and signaling adaptors, represents a rapidly expanding frontier that links epigenetic regulation to cellular metabolism and immune signaling in rheumatic diseases. A deeper investigation into this area is warranted and may reveal novel mechanistic insights and therapeutic opportunities.
